# Supplementary figures and images for: Overlapping functions of SIX homeoproteins during embryonic myogenesis
Source: PLoS Genet. 2023 Jun 2;19(6):e1010781. doi: 10.1371/journal.pgen.1010781 (PMC10266681; doi:10.1371/journal.pgen.1010781)

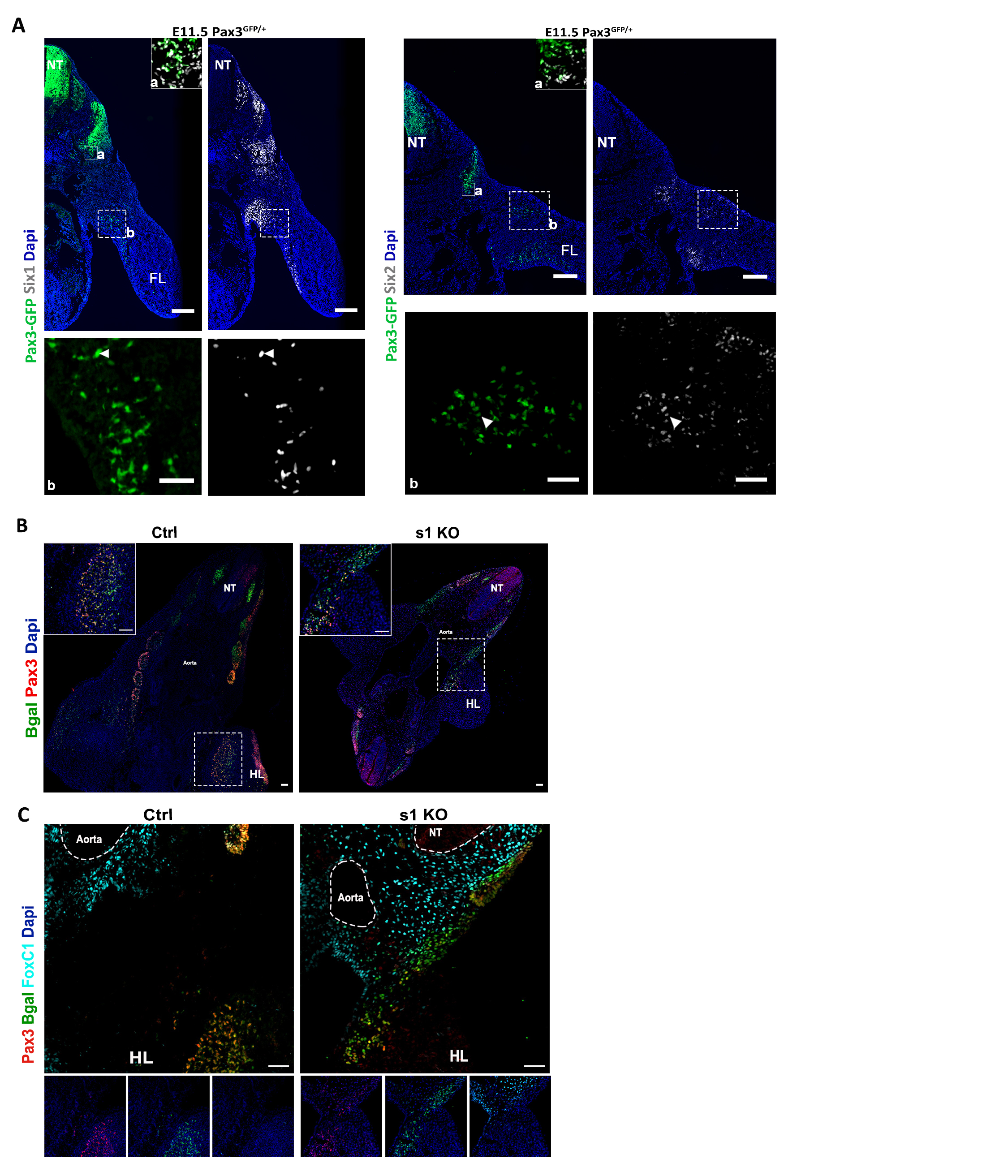

Supplement: S1 Fig — (A) Immunostaining on transverse sections of E11.5 Pax3GFP/+ embryos at the trunk level showing Pax3 (green), Six1 and Six2 (grey) and Dapi (blue); Upper panel “a” showing the mesenchymal cells and “b” showing the migrating progenitor cells to the limbs; FL: Forelimb, Sb = 200μm. Lower panel: zoom on the migratory progenitor cells “b”, Sb = 50μm. (B) Immunostaining on E10.5 Ctrl (s1 Hz) and s1KO (n = 1) embryos transverse sections at the limb buds level for ß-gal (green), Pax3 (red) and Dapi (blue); upper panel zoom represents the dashed white square; NT: Neural Tube, HL: Hindlimb; Sb = 50μm. (C) Immunostaining on E10.5 Ctrl (s1 Hz) and s1KO (n = 1) embryos transverse sections at the limb buds level for ß-gal (green), Pax3 (red), FoxC1 (cyan) and Dapi (blue). Lower panels represent a zoom of the Pax3 positive regions. HL: hindlimb, NT: neural tube; Sb = 50μm. (TIFF) [file pgen.1010781.s001.tiff]

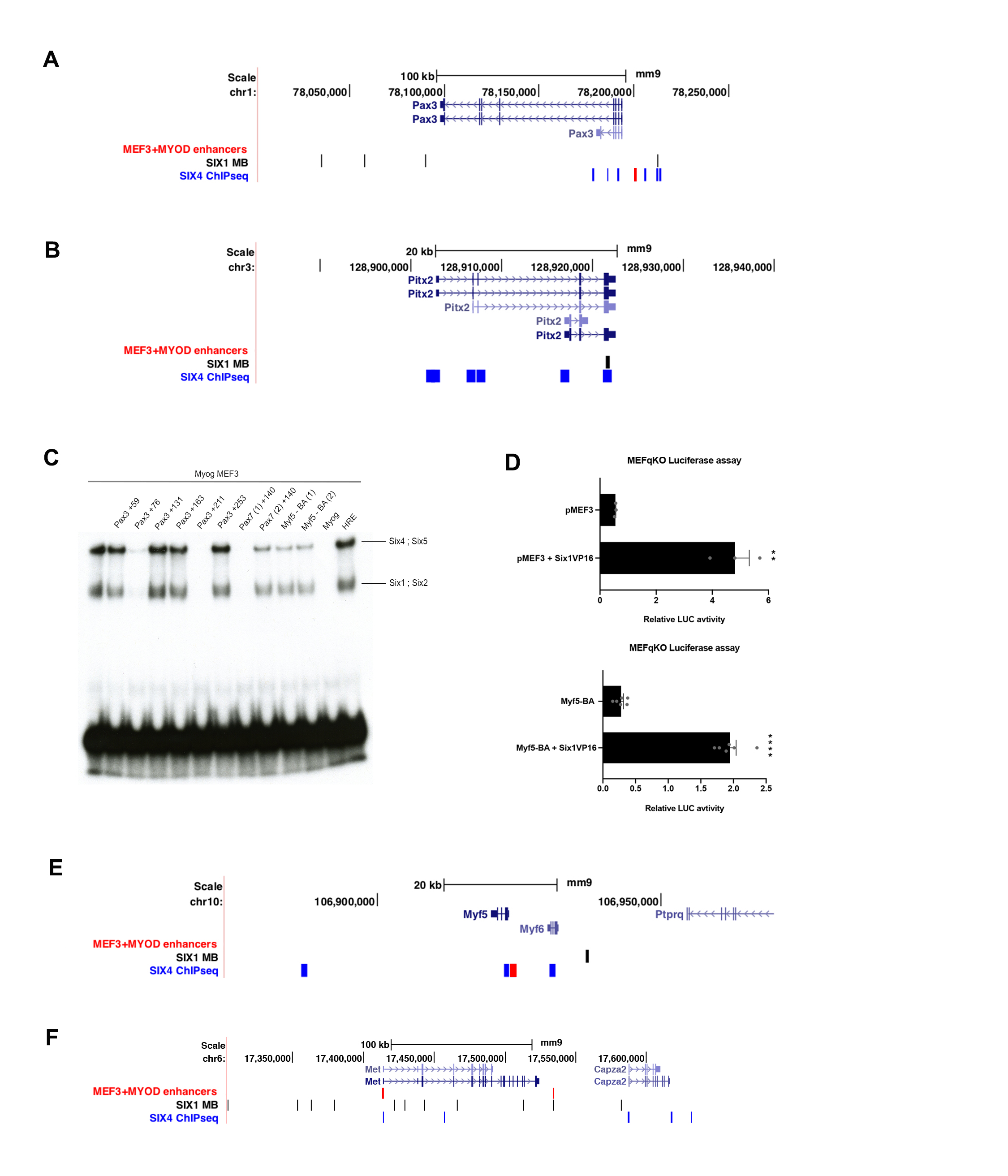

Supplement: S2 Fig — (A-B) Open chromatin regions shown by SIX1 and SIX4 ChIP-seq experiments at the Pax3 and Pitx2 regions, respectively. (C) Gel Mobility-Shift Assay (GMSA) using Myogenin MEF3 double stranded DNA probe with in vitro synthesized SIX1, SIX2, SIX4 and SIX5 and hundred-fold molar excess of indicated DNA competitor. See Table 2 for Pax3, Pax7, Myf5 and Myog DNA probes sequences. (D) Luciferase assays on mouse embryonic fibroblasts (MEF) qKO showing a significant activation of the poly-Mef3 promoter and of Myf5 branchial arch enhancer with Six1-VP16 chimeric protein; Statistical non-parametric t- test with mean ±s.e.m and **p<0.005, ****p<0.0001 (E-F) Open chromatin regions shown by SIX1 and SIX4 ChIP-seq experiments at the Myf5 and c-met regions respectively. Red rectangle for SIX4-ChIP-seq corresponds to Myf5 BA enhancer. (TIFF) [file pgen.1010781.s002.tiff]

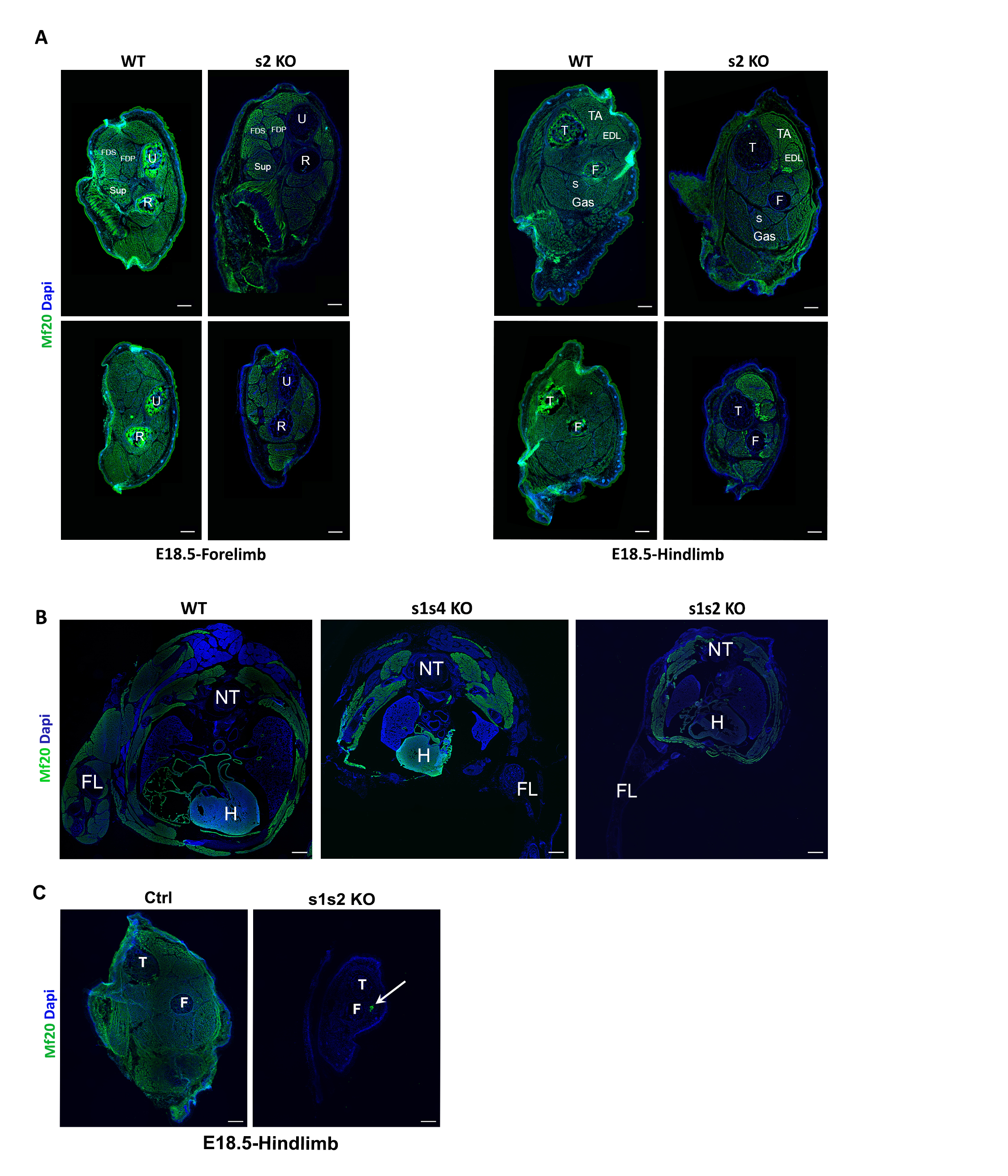

Supplement: S3 Fig — (A) Immunostaining on transverse sections of E18.5 WT and s2KO (n = 2) fetuses at the distal forelimb (left) Sb = 500μm and distal hindlimb (right) Sb = 500μm for sarcomeric myosins marked by MF20 (green) and Dapi (blue); U: Ulna, R: Radius, FDS: flexor digitorium sublimis, FDP: flexor digitorium profundus, Sup: supinator, F: Fibula, T: Tibia, TA: tibialis anterior, EDL: extensor digitorum longus, S: Soleus, Gas: Gastrocnemius. (B) Immunostaining on transverse sections of E18.5 WT, s1s4dKO (n = 3) and s1s2dKO (n = 2) fetuses at the trunk level for sarcomeric myosins marked by MF20 (green) and Dapi (blue); FL: Forelimb, H: Heart, NT: Neural Tube, Sb = 500μm. (C) Immunostainings on E18.5 Ctrl and s1s2dKO (n = 2) fetuses transverse sections on the distal hindlimbs level for sarcomeric myosins marked by MF20 (green) and Dapi (blue); T: Tibia, F: Fibula, Sb = 200μm. (TIFF) [file pgen.1010781.s003.tiff]

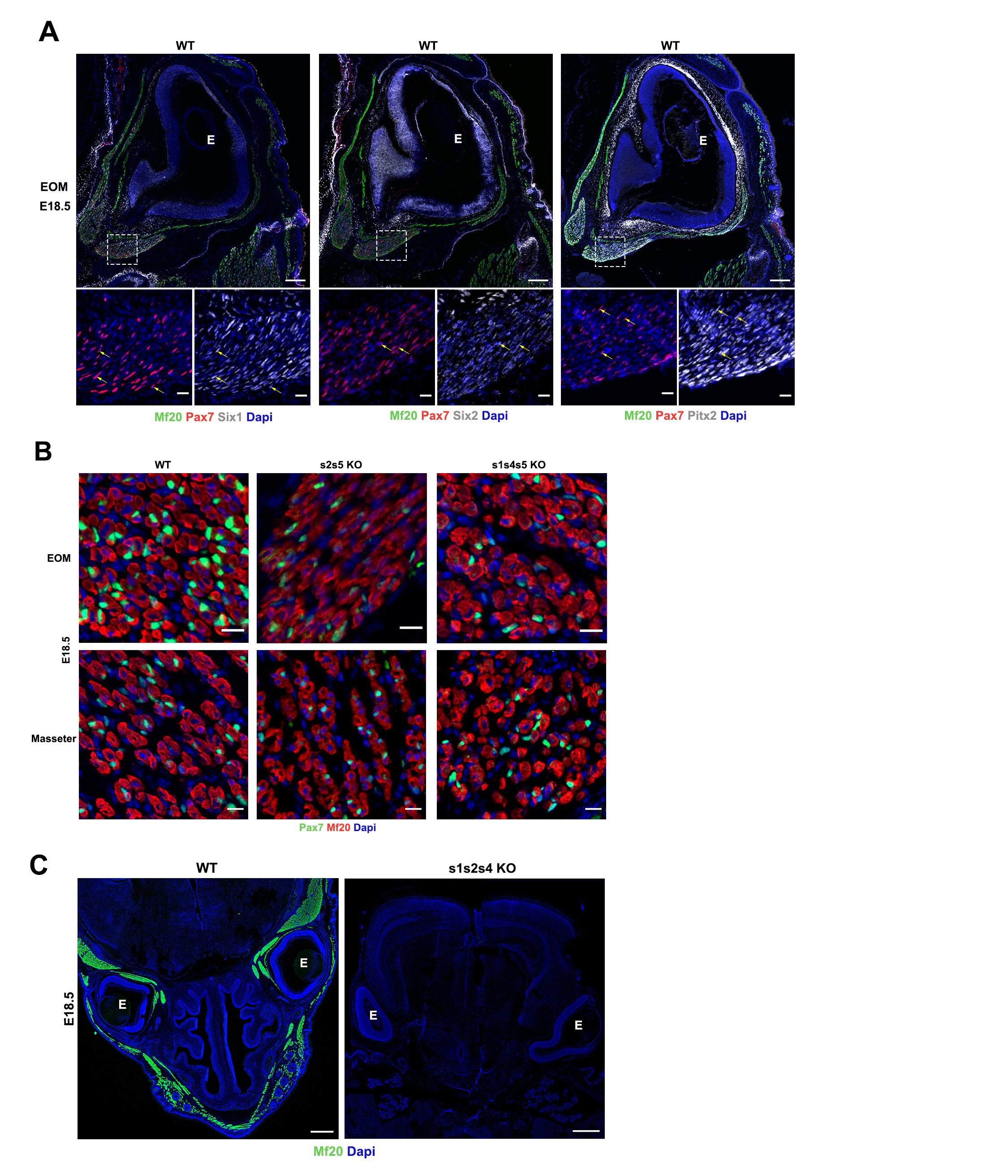

Supplement: S4 Fig — (A) Immunostainings on E18.5 WT fetuses on transverse sections at the head level showing the EOM marked by MF20 (green), and the Pax7 (red) cells expressing Six1 or Six2 or Pitx2 (grey) and Dapi (blue); E: eye, Sb = 150μm (upper panel) Sb = 20μm (lower panel). (B) Immunostainings on E18.5 WT, s2s5dKO (n = 2) and s1s4s5tKO (n = 2) fetuses at the head level showing the EOM (upper panel) and the masseter muscles (lower panel), Pax7 (green), MF20 (red) and Dapi (blue); Sb = 15μm. (C) Immunostainings on E18.5 WT and s1s2s4tKO fetuses on frontal sections at the head level showing the EOM marked by MF20 (green), and Dapi (blue); E: eye, Sb = 500μm. (TIFF) [file pgen.1010781.s004.tiff]

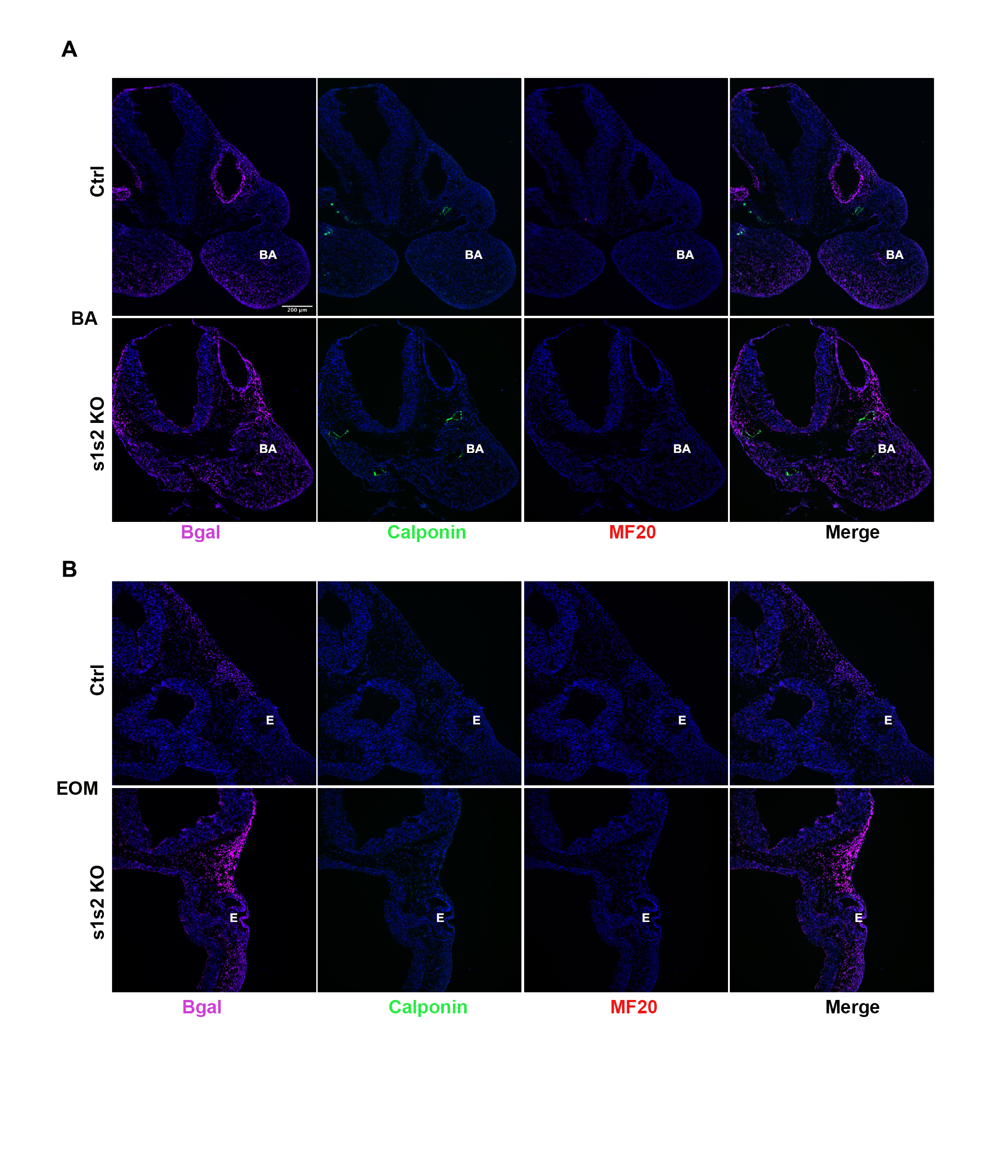

Supplement: S5 Fig — (A) Immunostainings on E10.5 Ctrl (s1 Hz), s1s2dKO (n = 3) embryos transverse sections at the branchial arches (BA) level for ß-gal (purple), Calponin (green), MF20 (red) and Dapi (blue); Sb = 200μm. (B) Immunostainings on E10.5 Ctrl (s1 Hz), s1s2dKO (n = 3) embryos transverse sections at the head (EOM) level for ß-gal (purple), Calponin (green), MF20 (red) and Dapi (blue); E: Eye, Sb = 200μm. (TIFF) [file pgen.1010781.s005.tiff]

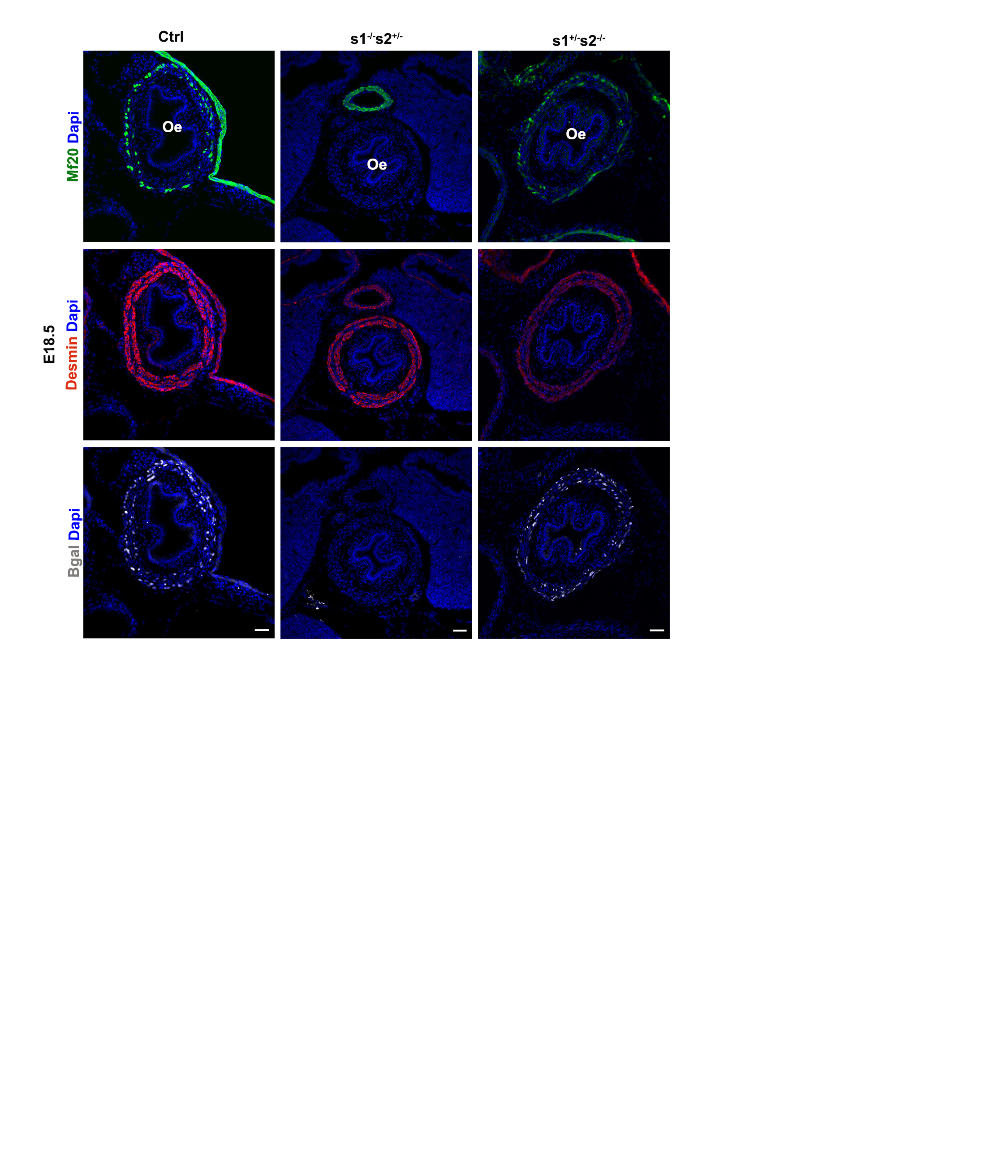

Supplement: S6 Fig — Immunostainings on E18.5 Ctrl (s1 Hz), s1KO::s2Hz (n = 2), and s1Hz::s2KO (n = 2) fetuses at the trunk level showing the esophagus stained with MF20 (green), Desmin (red) and ß-gal (grey); oe: esophagus, Sb = 40μm. (TIFF) [file pgen.1010781.s006.tiff]

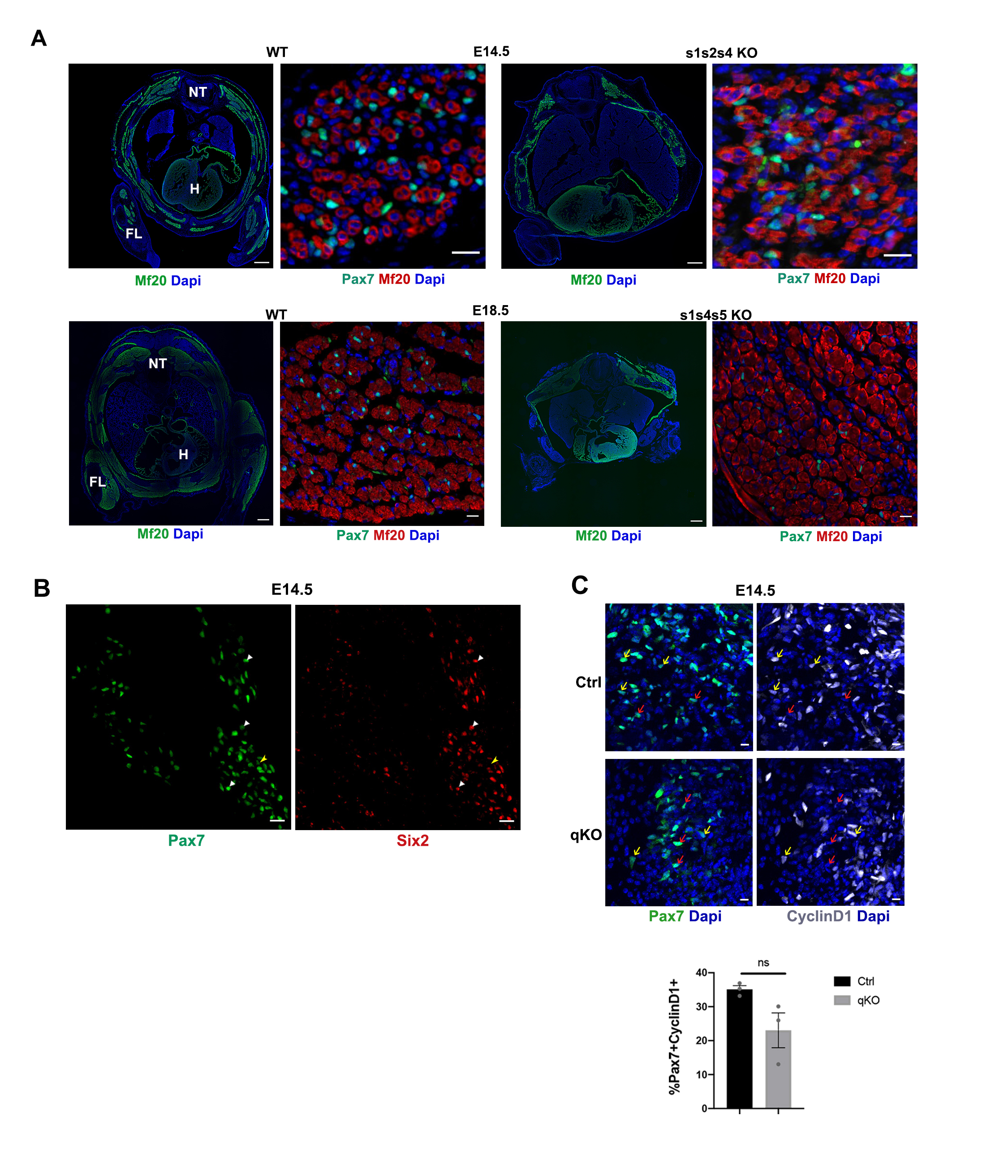

Supplement: S7 Fig — (A) Upper panel: immunostainings on transverse sections of WT and s1s2s4tKO E14.5 fetuses showing on the left the whole fetus with sarcomeric myosins marked by MF20 (green) and Dapi (blue); FL: Forelimb, H: heart, NT: Neural Tube, Sb = 300μm, and on the right a zoom on the dorsal muscle masses stained with Pax7 (green), MF20 (red) and Dapi (blue); Sb = 20μm. Lower Panel: immunostainings on transverse sections of WT and s1s4s5tKO E18.5 fetuses showing on the left the whole fetus with sarcomeric myosins marked by MF20 (green) and Dapi (blue); Sb = 300μm, and on the right a zoom on the dorsal muscle masses stained with Pax7 (green), MF20 (red) and Dapi (blue); Sb = 15μm. (B) Immunostainings on E14.5 WT fetuses transverse sections at the trunk level showing on dorsal muscle masses Pax7 (green) and Six2 (red); white arrowheads indicate Pax7+Six2+ cells and yellow arrowheads indicate Pax7+Six2- cells, Sb = 20μm. (C) Left: immunostainings of E14.5 Ctrl and qKO (n = 3) fetuses at the trunk level showing dorsal muscles masses staining for Pax7 (green), CyclinD1 (grey) and Dapi (blue); Sb = 10μm. Right: Quantification of the percentage of Pax7+ CyclinD1+ cells in the dorsal muscle masses of E14.5 Ctrl and qKO (n = 3) fetuses. Welch’s statistical test with mean ±s.e.m; ns: non-significant with p = 0.13. (TIFF) [file pgen.1010781.s007.tiff]

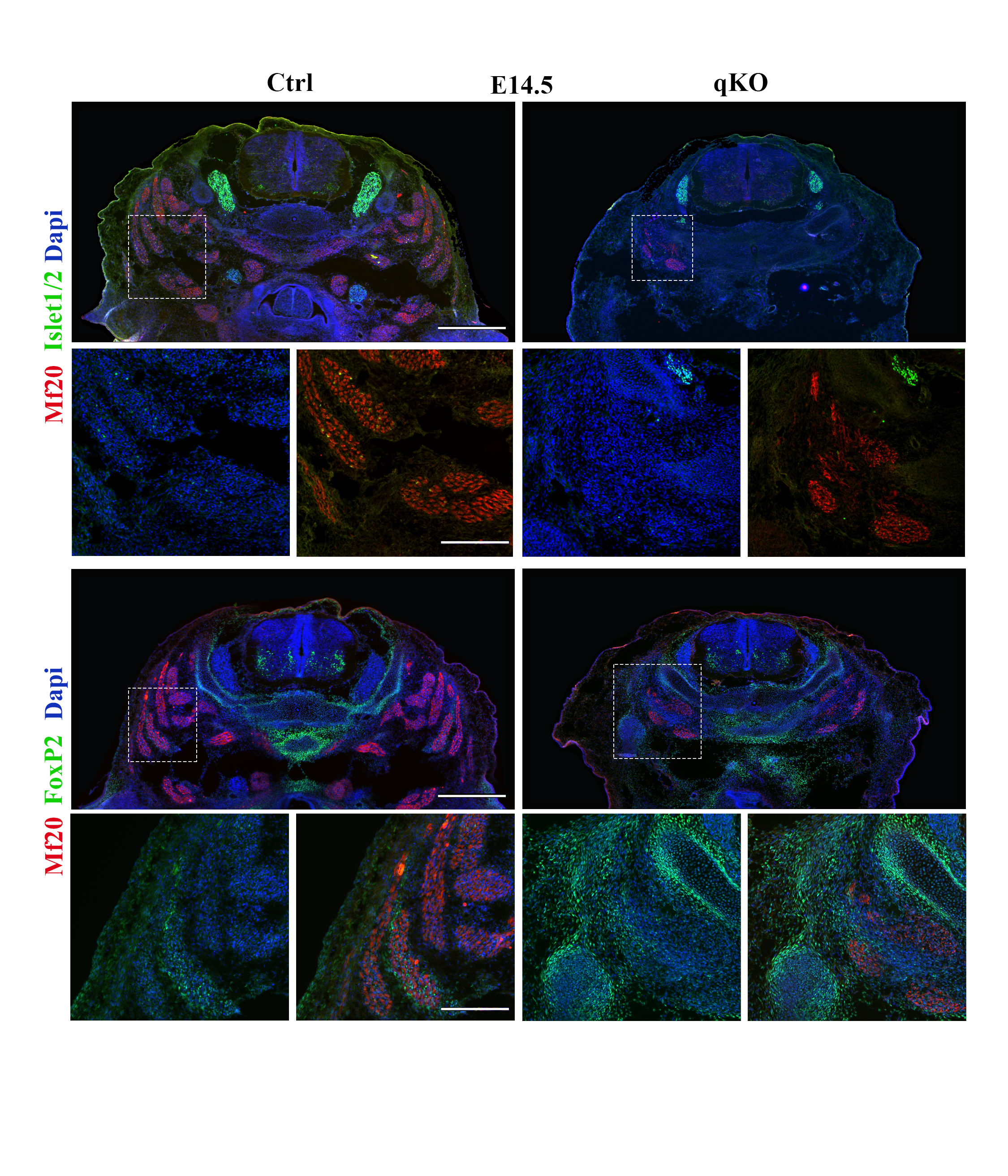

Supplement: S8 Fig — Immunostaining on a cross-section of E14.5 Ctrl and qKO fetuses at the trunk level showing MF20 (red), Islet1/2 and FoxP2 respectively (green) and Dapi (blue) Sb = 500μm. Zoom on the lower panels represent the dashed white squares and Sb = 200μm. (TIFF) [file pgen.1010781.s008.tiff]

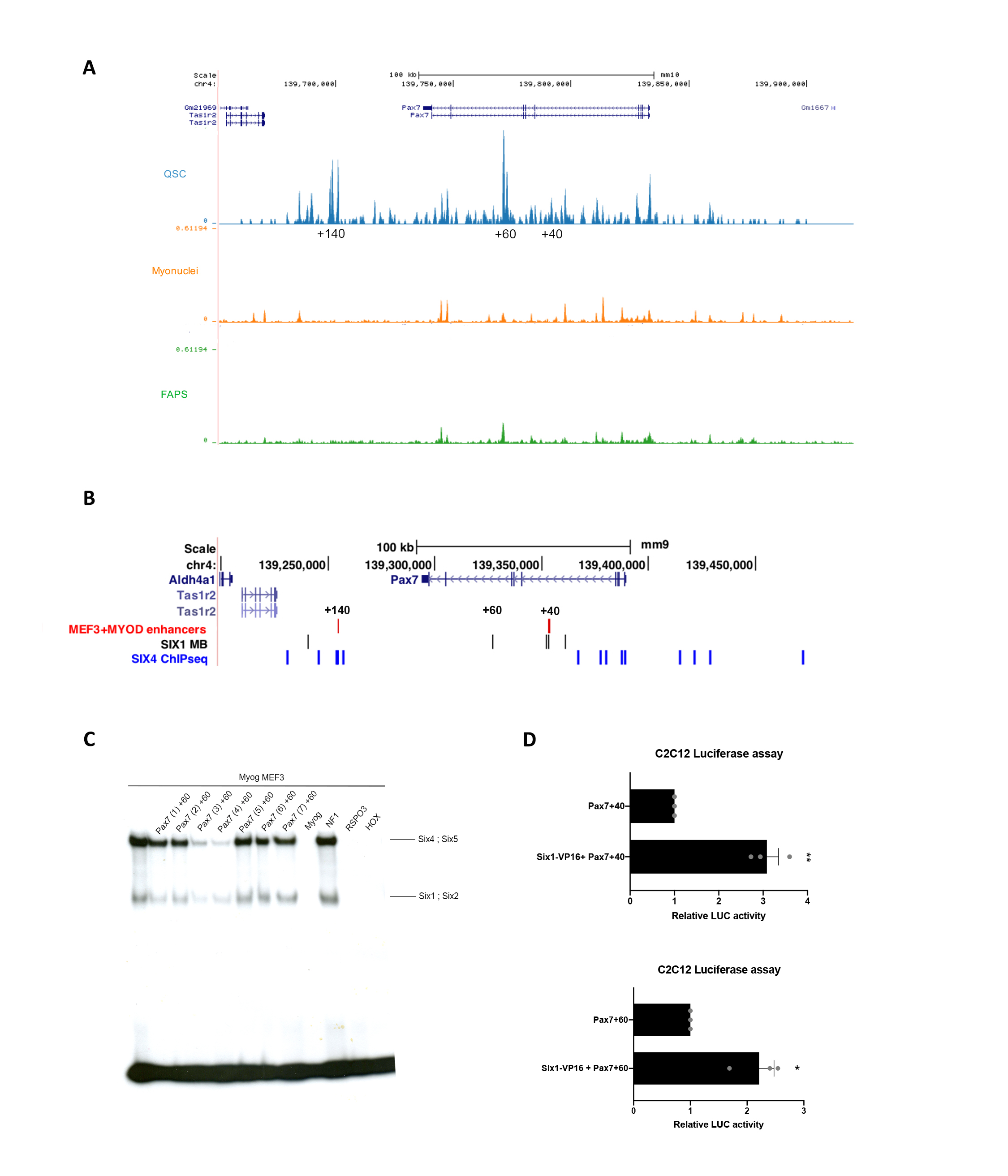

Supplement: S9 Fig — (A) snATAC-seq experiments with adult skeletal muscles at the Pax7 locus showing opening chromatin at +140, +60 and +40Kb regions. (B) Six1-ChIP-seq and Six4-ChIP-seq experiments at the Pax7 locus. (C) Gel Mobility-Shift Assay (GMSA) using Myogenin MEF3 double stranded DNA probe with in vitro synthesized SIX1, SIX2, SIX4 and SIX5 and hundred-fold molar excess of indicated DNA competitor. See Table 2 for Pax7, NF1, RSPO3 and HOX DNA probes sequences. (D) Luciferase assays in mouse C2C12 cells showing a significant activation of the Pax7+40 and Pax7+60 enhancers with Six1-VP16 chimeric protein. Statistical non-parametric t- test with mean ±s.e.m and **p<0.005 and *p<0.05. (TIFF) [file pgen.1010781.s009.tiff]

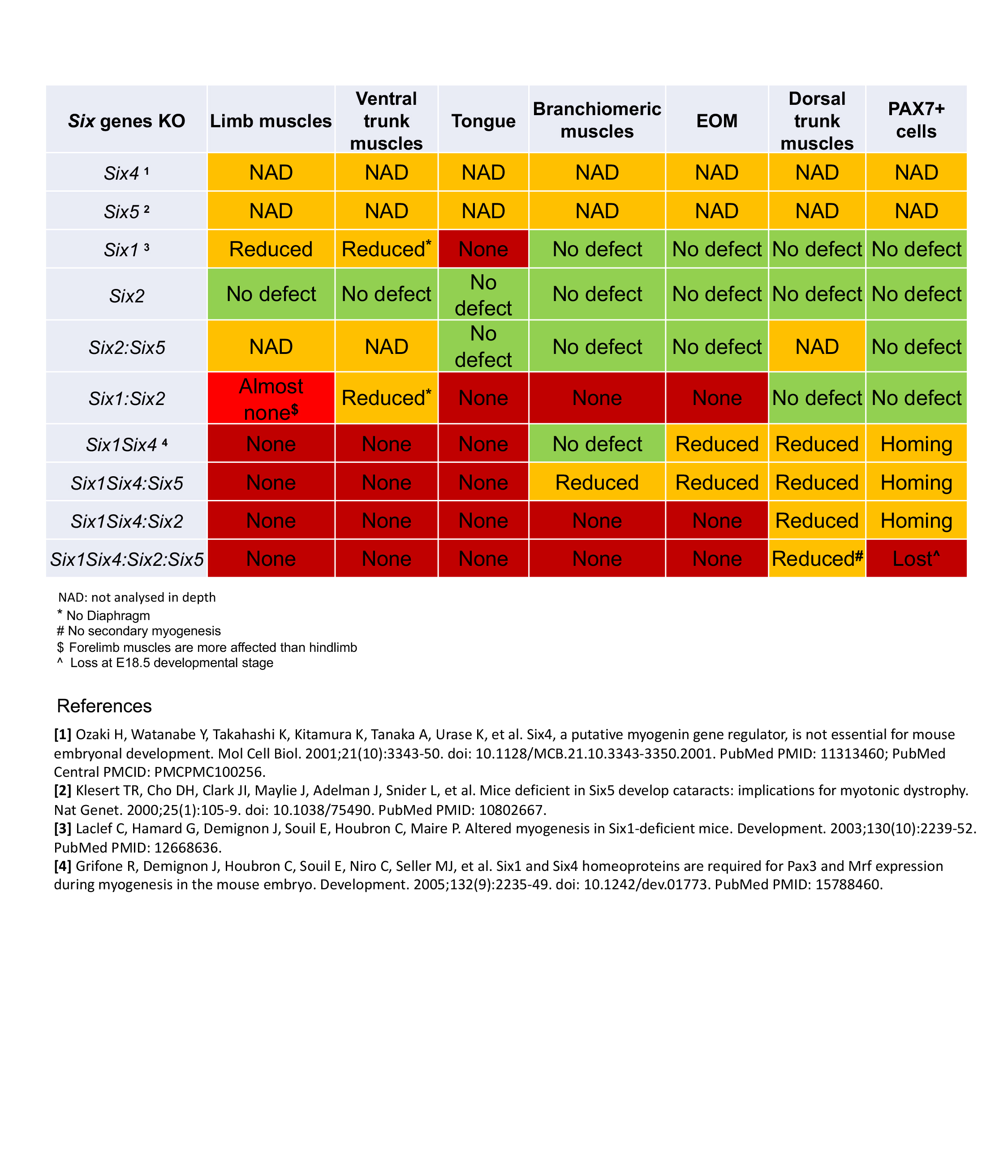

Supplement: S10 Fig — (JPG) [file pgen.1010781.s010.jpg]
