# Supplementary material for: Overlapping functions of SIX homeoproteins during embryonic myogenesis
Source: PLoS Genet. 2023 Jun 2;19(6):e1010781. doi: 10.1371/journal.pgen.1010781 (PMC10266681; doi:10.1371/journal.pgen.1010781)
Supplement: Attachment — Submitted filename: PLOS genet response to reviewers.pdf [file pgen.1010781.s011.pdf]

Wurmser et al.
Reviewer's Responses to Questions

The review is uploaded as an attachment.
Modifications introduced in the text appear in red.

Reviewer #1:
We thank the reviewer for his/her helpful comments.

For example, SIX are involved in craniofacial myogenesis, and Myf5 expression fails to be activated. The alternative fate of the cells should be characterised further (page 7, Fig. 2B). The authors suggest they may become smooth muscle cells, this could be assessed using relevant markers.
We thank the reviewer for his/her suggestions. In the course of better identifying the behavior of S1S2 and S1S2S4 mutant cells at the branchial arch and EOM levels we failed to observe Calponin expression suggesting that these cells are not rerouted to the smooth muscle fate. We corrected the text and do not claim anymore that they adapt a smooth fate at the craniofacial level. What we show is that myogenic fate acquisition is blunted in absence of Six1/Six2 due to the absence of Myf5 gene expression. RNAseq experiments with mutant BAs LacZ+ cells should be performed to formally answer to the behavior of mutant cells, experiment we have not been able to perform.

The also find that quadruple KO fetuses do form neck muscles and myogenesis is activated in this context. There is no functional explanation for this important difference.
We showed that epaxial myogenesis takes place until E14.5 and that few neck muscles are also formed. We suggest that these neck muscles have a somitic origin because we were unable to detect FOXP2 or Isl1/2 expression in these neck muscles at E14.5, while FOXP2 and Isl1/2 positive neck muscle masses can be observed in wt embryos at that stage. FOXP2 is suspected to be specific of craniofacial mesodermal cells derivatives. We observed FOXP2 cells in some neck muscles in wt E14.5 embryos. As remaining neck muscles of qKO embryos are FOXP2- , we propose that these remaining muscles have a somitic origin. We failed to detect PAX3+ myogenic stem cells in E14.5 wt or mutant neck, excluding the possibility to use this marker at E14.5 to identify more robustly their embryonic origin. We argue that this remaining epaxial/neck myogenesis takes place in absence of SIX proteins, reminiscent of the remaining myogenesis observed in Pax3/Pax7 double mutant embryos. We did not explore further how MRF gene expression can be activated in absence of Six/Pax genes but explain that the early Myf5 expression is dependent on LEF/GLI proteins and Notch signaling. It is nevertheless possible that other transcription factors that LEF/GLI are required to induce the formation of the first myogenic cells, those remain to be identified. SnRNA-seq with mutant samples may identify those transcription factors expressed in in qKO myonuclei.

There are fewer fibres and epaxial muscle mass does not increase. A possible mechanistic explanation suggested are effects on Pax7 enhancer elements identified previously by the lab using snATAC seq. In vitro GMSA and transient transfection of reporters in C2C12 cells support a possible interaction with a constitutively active SIX-VP16 protein. Effects on the expression of the Pax7-GFP transgene (BAC) could have been examined in vivo in the context of various SIX KOs to confirm the cell-based experiments.

We agree with the reviewer that study of Six1$^{-/+}$Six4$^{-/+}$Six2$^{-/+}$Six5$^{-/-}$::Pax7-nGFP and Six1$^{-/-}$Six4$^{-/-}$Six2$^{-/-}$Six5$^{-/-}$::Pax7-nGFP embryos would have been relevant, but we think that the absence of PAX7+ cells observed in E18.5 qKO provides a strong argument to claim that Pax7 expression is no more detected in muscles masses of qKO and that its expression may be under the control of SIX proteins. The GMSA/Sixchip-seq/ATAC-seq data argue that SIX proteins are able to bind to several opened DNA regions at the Pax7 locus in Pax7+ adult stem cells, the transfection experiments that SIX may modulate Pax7 gene expression, and the analysis of Six mutant embryos that Six are required to maintain Pax7 gene expression in embryonic myogenic stem cells. We have not been able to generate and analyze Six1$^{-/+}$Six4$^{-/+}$Six2$^{-/+}$Six5$^{-/-}$::Pax7-nGFP in the course of this reviewing process.

Based on immunostaining and cell counts the authors suggest that PAX7 positive cells differentiate and thus the progenitor population becomes depleted. This is very difficult to see in the panels and not convincing.

We thank the reviewer for addressing this issue, we hope that the new figures with the added arrows will allow to detect easier the different subpopulations of cells in parallel with their quantification and convince the reader regarding the behavior of mutant myogenic stem cells that we explain.

Reviewer #2:

We thank the reviewer for his/her enthusiasm concerning our manuscript.

1. In Fig. S1B it is very hard to appreciate normal muscle patterns in Six2 KO fetuses in the images provided. Sections of comparable levels and orientation should be shown. Also, it would be nice to label the muscles.

We corrected the Fig S1B (now Fig S2) , and now label a few muscles.

2. Since Six2 is also expressed locally in limb mesenchyme, smaller phenotypes caused by local mesenchyme-myoblast interactions (as e.g. in the Tbx3 KO with only few muscles affected, DOI: 10.1242/dmm.025874) may have been overlooked. Ideally, ideally several sections at different levels should be shown to detect such changes. However, I appreciate that this is not the focus of the manuscript

We now show sections at two hindlimb and forelimb levels, and label a few muscles. We found no missing muscles. We agree with the reviewer that early expression (E9.5-E10.5) of Six2 is not detected in hypaxial myogenic progenitors. Later on, Six2 can be detected in migrating limb myogenic stem cells and in other cell types of the limb. The analysis of the Six2 KO at distal hindlimb or forelimb levels shows no major muscle hypoplasia, arguing that if the local interplay between mesenchyme-myoblasts is compromised in Six2 mutants, its absence nevertheless does not lead to severe muscle defects in terms of myofiber number and muscle patterning. We do not provide in this study a precise kinetic of SIX2 expression in the different cell types present in the growing limb bud, and cannot exclude specific defects in tendons formation for example, as suggested by Six2 expression pattern (doi: 10.1242/dev.121.3.693, DOI: 10.1016/j.ydbio.2013.02.023**).**

3. The authors claim (p6) that "we observed … few Six1-β-Gal+ s1s2 mutant cells at the dorsal aorta level, expressing α-SMA or CD31", which likely is supposed to be compared to many of such cells in s1/s4 DKOs; however, on the images in Fig. 1A, I do not see differences between s1/s4 or s1/s2 embryos. **A quantification should be provided.** Along the same line, the authors find "few PAX3+ cells in limbs of s1/s2 embryos; are these indeed fewer than in controls, this **should be quantified.** Are the sections shown in Fig. 1B on fore- or hindlimb levels? **Both should be shown**, as muscle formation in forelimbs appears abrogated, while being affected less severely in hindlimbs (although this is not properly displayed; sections as in Fig. S1C should be shown for the hindlimb level as well to appreciate proximal muscles).

We now provide quantification, as suggested by the reviewer, these reinforce the data.

4. In s1/s4 DKOs the authors have demonstrated previously lack of c-met expression; for the sake of completeness, the authors may want to show this for the s1/s2 mutants as well.

We ordered two c-Met antibodies, but unfortunately were not able to detect specific c-Met expression in the wt embryo at the hypaxial level. We agree with the reviewer that this information would have been interesting to provide.

5. Fig. S3B and Fig. 5B should be quantified

We quantified the results of Fig5B. We did not quantify the Fig. S3B (now Fig.S4B) because we analyzed only two Six2Six5 and two Six1Six4Six5 mutants at several levels. We present quantification when at least three distinct samples have been analyzed. For

Six2Six5 and Six1Six4Six5 mutants we only revealed the presence of myogenic stem cells in the muscle masses of the corresponding mutant fetuses, as compared with their absence in qKO. We cannot conclude in these mutants regarding the interplay between the mutant myogenic stem cells, and their environment as we did already in the Six1Six4 mutant (Wurmser et al, 2020).

6. In Fig. 3B, the authors use Desmin expression as an indicator of a possible switch to a smooth muscle fate of EOM muscle progenitors; it would be more appropriate to use alpha-SMA immunostaining as the authors have used in the trunk.

We thank the reviewer for his/her suggestion. We used Calponin and alpha SMA immunostaining to better illustrate the cell fate modification of mutant samples, but were unable to detect Calponin and alpha SMA signal. We re-write this aspect of our work.

Reviewer #3:
We thank the reviewer for his/her helpful comments.

Precisely; in Figure 1C, the authors claim that "An increased FOXC1 expression was also detected in mutant b-Gal+ hypaxial cells of the dermomyotome of E10.5 s1s2dKO at the hindlimb level, where a few of those b-Gal+ s1s2dKO cells expressed FOXC1". Such statement could be supported by a quantification of the number of FOXC1 positive cells in the dermomyotome, as well as a quantification of the number of FOXC1+/β-Gal positive cells in Ctrl, s1s4 KO and s1s2 KO samples.
We now present a quantification of the data shown in Figure 1 concerning FOXC1 expression.

In Figure S3B, the authors claim that "While the masseters, derived from the first branchial arch, formed normally in s1s4dKO, they were hypoplasic in s1s4s5tKO. Furthermore, both s1s4s5tKO and s2s5 dKO muscles masses show the presence of less PAX7+ cells (Fig.S3B). There is no data to support the hypoplasia of the Masseter in s1s4s5tKO, and the assumption that the number of Pax7+ cells is reduced is not supported by quantitative data. Furthermore, the panels of immunostaining for Pax7 in the Masseter of WT, s2s5 KO and s1s4s5tKO is not convincing enough to make the above statement.
We thank the reviewer for his/her suggestions. In Figure 2A, we show (N=2 animals, n>3 sections/animal) that the masseter of 18.5 Six1Six4Six5 mutant is hypoplasic; the number of myofibers at the masseter level is drastically reduced. We deleted the sentence concerning the lower number of myogenic stem cells associated with remaining masseter mutant myofibers, and only indicate now that PAX7 myogenic stem cells could be detected at the masseter and EOM levels in these mutants.
As answered for reviewer 2: We did not quantify the Fig. S3B (now Fig. S4B) because we analyzed only two Six2Six5 and two Six1Six4Six5 mutants at several levels. We present quantification when at least three distinct samples have been analyzed. For Six2Six5 and Six1Six4Six5 mutants we only revealed the presence of PAX7+ myogenic stem cells in the muscle masses of the corresponding mutant fetuses, as compared with their absence in qKO. We cannot conclude in these mutants regarding the interplay between the mutant myogenic stem cells, and their environment as we did already in the Six1Six4 mutant (Wurmser et al, 2020).

In Figure 5B, the authors mention that "As suspected, qKO fetuses showed an increase in embryonic MYH (MYH3) as well as slow MYH (MYH7) positive myofibers, while no fast perinatal MYH (MYH8) was detected in their remaining epaxial muscle masses (Fig. 5B). A quantification of the number of positive fibres could be carried out to support this statement.
We now quantified the results presented in the Figure 5B.

In Figure S5C, the authors declare "Accordingly, we observed a decreased percentage of PAX7+ CyclinD1+ cells in the qKO E14.5 fetuses in epaxial muscles (Fig.S5C)." The graph shows a trend, however, the quantification of the number of Pax7+ CyclinD1+ cells in the qKO versus control does not indicate statistical significance. Could the author provide the p value? Could they strengthen this data with a staining for Ki67, as they did in the final figure?
We now indicate the p-value -0.13- for PAX7+CyclinD1+ cells in the legend of the figure.

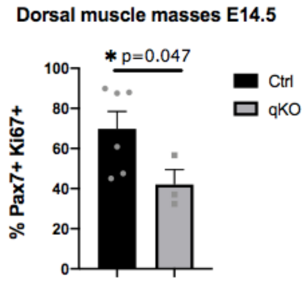

The results for the quantification of PAX7+Ki67+ cells presented in Figure 5 in a pie chart are shown here as a graph for the reviewer to get a better idea.

The role of Six5 remains elusive and could be more broadly discussed. Have the authors investigated the expression of Six5 in the presomitic mesoderm? The authors have shown in this study and previously that a proportion of Six1 and Six2 cells are co-expressing Pax3 in the dermomyotome, what about the expression of Six5 in this compartment?

The expression pattern of Six5mRNA or SIX5 protein is still lacking, but Yajima et al (2010) showed Six5 expression in PAX7+ myogenic stem cells. We addressed this question when SIX5 antibodies were available (gift from Dr *Charles Thornton*) and observed that SIX5 and SIX4 are detected in the PSM and somitic dermomyotome (see Figure below), while we did not detect SIX1 nor SIX2 in the PSM. That is the reason why we contacted S.Tapscott to analyze the phenotype of Six5 mutant mice. We failed to detect major defects in those mutant and even Six1-/+Six2-/+Six4-/+Six5-/- are viable and fertile. Six5 is nevertheless involved with Six4 in ventral body wall closure (doi: 10.1242/dmm.034611**). SIX5 antibodies are no more available, and we published already the coexpression of SIX5 with Myogenin in the human embryo (Fougerousse et al, 2002). The precise spatiotemporal expression of SIX5 protein during embryogenesis remains to be established, we tried to perform a long time ago in situ hybridization, but it seemed that Six5 mRNA does not accumulate to high levels, and it was difficult to detect it, probably explaining the lack of convincing data concerning its expression. We introduced one sentence in the introduction concerning SIX5 and myogenesis.**

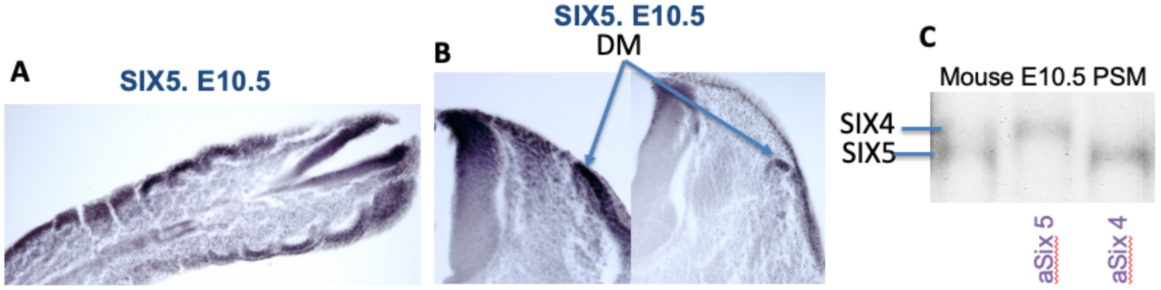

A, B: SIX5 proteins are detected in PSM and in dermomyotomes (DM) of E10.5 mouse embryos with specific SIX5 antibodies.

C, GMSA with a Myogenin MEF3 probe and nuclear extracts from PSM of E10.5 embryos and showing the binding of SIX4 and SIX5 proteins. Addition of SIX5 antibodies aSix4 reveal the binding of SIX4, while addition of SIX4 antibodies (aSix4) reveal the binding of SIX5 to MEF3 DNA probe.

To help recapitulate their findings, the authors could generate a schematic that illustrate the different roles of Six genes in myogenesis, confirmed and presumed by this study.

We thank the reviewer of his/her suggestions. We now provide two new figures to recapitulate our findings (Fig.7 and S10).
